# Will the Debate Over e-Cigarettes Start Cooling Down?

**DOI:** 10.1001/jamanetworkopen.2018.5945

**Published:** 2018-12-07

**Authors:** Theodore L. Wagener

**Affiliations:** **Author Affiliations:** Department of Pediatrics, University of Oklahoma Health Sciences Center, Oklahoma City; Oklahoma Tobacco Research Center, Stephenson Cancer Center, Oklahoma City.

The cost-benefit of electronic cigarettes (e-cigarettes) continues to be debated in the public health literature. The most recent estimates indicate that 10.8 million US adults (4.5%) have used an e-cigarette in the past 30 days.^1^ Among high school students and young adults (aged 18-24 years), these rates are even higher, at 1.73 million (11.7%) and 2.8 million (9.2%), respectively.^2^ However, cigarette smoking rates continue to decline, and this may be due, at least in part, to smokers switching from smoking cigarettes to using e-cigarettes (ie, vaping). Indeed, most e-cigarette users are current or former smokers. The potential promise of e-cigarettes as a public health benefit lies in their ability to serve as a sufficient replacement for smoking while also reducing users’ exposure to harmful toxicants, rendering them significantly less harmful than cigarettes. While few randomized clinical trials have examined the efficacy of e-cigarettes as a replacement for combustible cigarettes,^3^ with less-than-encouraging outcomes, studies examining changes in smokers’ exposure to tobacco toxicants on switching to e-cigarette have shown more promise but have often been limited in scope. More comprehensive studies examining e-cigarette users’ exposure to harmful toxicants, especially compared with smokers and nontobacco users, is needed.

Goniewicz and colleagues^4^ begin to address this need, conducting what appears to be to date the most comprehensive study in terms of the range and number of tobacco-related biomarkers examined among e-cigarette users. Specifically, using data collected from Wave 1 (2013-2014) of the Population Assessment of Tobacco and Health Study, a nationally representative, longitudinal cohort study designed to assess tobacco use and health, the authors examined and compared the levels of tobacco toxicant exposure among a sample of never tobacco users, exclusive users of e-cigarettes, exclusive users of cigarettes, and users of both cigarettes and e-cigarettes (dual users).

Largely consistent with findings from previous smaller, less-comprehensive studies, Goniewicz et al^4^ showed that, although exclusive e-cigarette users had significantly higher levels of exposure to nicotine and other tobacco-related toxicants than never users, they had significantly lower levels of exposure compared with exclusive smokers. Dual users evidenced the highest levels of tobacco toxicant exposure, even more than exclusive smokers. While likely influenced by smoking frequency, this finding has also been consistent in the literature and is cause for concern as most e-cigarette users are dual users.^1^ Although some dual users may be on the path to completely switching, it is also likely that some may be failed switchers or long-term dual users who use e-cigarettes when they cannot smoke. If long-term dual use is more common than complete switching and exposes users to even greater amounts of tobacco toxicants, what may be the downstream health consequences of this type of use? Clear health messages must be delivered to smokers that completely switching from smoking to e-cigarette use is necessary to confer a significant reduction in exposure to cardiovascular, respiratory, reproductive, and development toxicants, as well as carcinogens.

While touched on briefly by Goniewicz and colleagues,^4^ another point of consideration is that, given the timeframe of data collection for this study (2013-2014), it is likely that many of the e-cigarettes being used were early-generation devices, which have been shown to have inefficient nicotine delivery. Since that time, e-cigarettes have continued to evolve, with newer-generation products demonstrating significantly improved nicotine delivery, and maybe addiction potential, often due to increased power (ie, wattage).^5^ However, with increased power comes increased heating of the e-liquid and the potential for greater thermal degradation of nonnicotine compounds, which are largely responsible for e-cigarette–produced toxicants. Increased power has been directly implicated in raising the production of carbonyl compounds, such as formaldehyde,^6^ a toxicant not examined in the present study. Moreover, increased power also leads to significantly greater production and consumption of e-cigarette aerosol (vapor) among users,^5^ likely increasing users’ exposure to toxicants. With continuous e-cigarette product evolution, it remains to be seen how the toxicant exposure presented in this study will compare with future examinations of exclusive e-cigarette users in the next wave of Population Assessment of Tobacco and Health data.

One of the newest and most popular e-cigarettes on the market, JUUL, garnering over 70% of the total US e-cigarette market share, is successfully bucking this high-wattage trend. This product uses a combination of low power but high concentrations of nicotine salts in its e-liquid, mixed with benzoic acid to lower the pH. Anecdotally, this combination improves the sensory experience, making it easier to inhale but still delivering what patent documents demonstrate as cigarette-like levels of nicotine.^7^ This combination of high nicotine but low wattage may be ideal to reduce nonnicotine exposures to tobacco-related toxicants—high levels of nicotine to reduce the number of puffs needed to achieve satisfying nicotine levels and low power to reduce the amount of toxicants produced.

On the other hand, JUUL’s ease of use and discrete, high-tech styling (resembling a USB drive) appear to be attractive to youth and young adults,^8^ potentially leading to a surge in youth who never smoked starting to use e-cigarettes. What is more, even before the emergence of this product as a market leader, more than half of current e-cigarette users were younger than 35 years, of whom 44% reported never smoking cigarettes.^1^ If this trend continues, e-cigarettes may no longer just have to be less harmful than cigarettes to convince regulatory officials of their benefit to the public’s health.

What does the future hold for e-cigarettes? With minimal regulation the evolution will continue. Manufacturers of e-cigarettes will produce products with improved performance, increased quality, lower cost, and more attractive marketing. The inevitable outcome of this evolution is that products will become more appealing and addictive with greater reach. Hopefully, they will also become safer, but only if the market or regulations demand it. More appealing, addictive, and affordable products will likely be even more competitive with combustible cigarettes, having the best chance of helping smokers switch to a less-harmful alternative. However, more appealing, addictive, and affordable products will also be the most appealing to youth, introduce youth to nicotine, and sustain nicotine addiction. How will we choose? Is the debate cooling down? I think it is only heating up.
